# Corrosion Resistance and Thermal Conductivity Enhancement of Reduced Graphene Oxide–BaSO_4_–Epoxy Composites

**DOI:** 10.3390/polym14153144

**Published:** 2022-08-02

**Authors:** Tung-Yuan Yung, Wen-Fang Lu, Kun-Chao Tsai, Jeng-Shiung Chen, Kwan-Nang Pang, Yu-Chih Tzeng, Hsin-Ming Cheng, Po-Tuan Chen

**Affiliations:** 1 Nuclear Fuels and Materials Division, Institute of Nuclear Energy Research, Taoyuan 325, Taiwan; tyyung@iner.gov.tw (T.-Y.Y.); wflu@iner.gov.tw (W.-F.L.); tsaijohn@iner.gov.tw (K.-C.T.); 2 Yottadeft Optoelectronics Technology Co., Ltd., Taipei 10460, Taiwan; jsc@yottadeft.com; 3 Institute of Earth Science, Academia Sinica, Taipei 10591, Taiwan; knpang@earth.sinica.edu.tw; 4 Department of Vehicle Power System Engineering, Chung Cheng Institute of Technology, National Defense University, Taoyuan 335, Taiwan; a0932467761@gmail.com; 5 Department of Electronic Engineering, Organic Electronics Research Center, Ming Chi University of Technology, New Taipei City 243, Taiwan; smcheng@mail.mcut.edu.tw; 6 Department of Vehicle Engineering, National Taipei University of Technology, Taipei 106, Taiwan

**Keywords:** graphene, epoxy, composite, corrosion resistance, thermal conductivity

## Abstract

The results of studies on the corrosion protectiveness and thermal conductivity of reduced graphene oxide–BaSO_4_ epoxy composites are reported here. A commercial epoxy resin and reduced graphene oxide (rGO) were blended with a hardening reagent and then mixed with prepared BaSO_4_–epoxy resin (B–epoxy). The reduced graphene oxide–BaSO_4_–epoxy composite (rGO–B–epoxy) paste was used to coat the surfaces of Al 7205 alloy and the corrosion and thermal properties were investigated. A corrosion test in a 3.5 wt% synthetic sea water solution showed that the composite coating containing BaSO_4_ had the best corrosion resistance. Moreover, the rGO–B–epoxy composite showed better protection against corrosion than the epoxy alone. The rGO–B–epoxy composite with 5 wt% BaSO_4_ had an in-plane coefficient of thermal conductivity of approximately 165.0 W/m K, and the in-plane thermal diffusivity was 71.38 mm^2^/s. In standard thermal conductivity tests, all three samples had values below 40 W/m K. The rGO–B–epoxy composites showed good surface corrosion protection and in-plane thermal conductivity.

## 1. Introduction

Graphene, as a new high-profile two-dimensional (2D) material, has shown promising application potential because of its prominent thermal [1,2,3], mechanical [4,5], anticorrosive [6,7], electrochemical [8,9], optical [10,11], and electric [12,13] properties. The largest amount of published research in this area has involved the mechanical properties of graphene–polymer composites [14].

Although graphene should ideally be a single layer of carbon atoms for honeycomb lattices, because of its unstable thermodynamics, this single layer is likely to form multiple layers of graphene or a graphite-like material with greater stability and lower surface energy. The functional groups could help the interaction with the polymer matrix or others molecules. The reduced graphene oxide with certain surface functional groups would be formed during the chemical reduction procedures. The structure of graphene is easily formed by a layer-by-layer structure; the delocalized electrons in sp^2^ hybrid orbital form pi–pi stacking. This causes problems for the dispersion of the graphene in a solution or polymeric matrix [15,16]. It is reasonable to use graphene as a 2D material to fill in a polymer matrix to enhance the mechanical properties of a composite in traditional polymer composite applications. A small amount of graphene can dramatically enhance the mechanical, electrical, and biological properties of a material [17]. Of course, adding a larger amount of graphene may not be the best method because of the dispersion issue [18]. Reduced graphene oxide (rGO) is a practical substitute for graphene that can be applied to a polymer resin to enhance its chemical and mechanical properties with the surface functional groups, such as carboxy, epoxyl, and hydroxyl groups. The graphene or rGO play almost the same roles for mechanical and electronic or thermal properties in polymer composites. Generally speaking, the wet chemical methods for synthesizing graphene may be called reduced graphene oxide.

Epoxy resin is a thermosetting polymer that is used for high-performance applications, with properties such as toughness, heat resistance, chemical resistance, and adhesion. Graphene–epoxy or rGO–epoxy composites have been used in new and interesting material applications for advanced technology [19,20]. The addition of a small amount of graphene or rGO (approximately 1 wt%) can enhance the mechanical properties of graphene/rGO-reinforced epoxy resin composites. One published study showed that graphene–epoxy composites with 0.5 wt%, 1 wt%, and 1.5 wt% graphene increased the Young’s modulus from 0.675 to 8 Gpa. The 0.5 wt% graphene–epoxy composite, compared to pure epoxy resin, was an improvement in Young’s modulus of about 91.6% [14].

The poor alignment/dispersion of graphene/rGO in a polymer matrix can have a negative impact on the expected properties of graphene/rGO–epoxy composites. Various dispersion methods are applied in the preparation of polymer resins with graphene/rGO. The pi–pi stacking of graphene/rGO is a driving force for aggregation. The dispersion and stability of the coating paste are the crucial issues for polymer composite applications. A better alignment for the graphene/rGO results in a higher Young’s modulus. Greater interaction between the polymer matrix and graphene/rGO results in better tensile and elongation properties. Greater interfacial interaction produces better enhancement of the mechanical, electrical, and thermal properties [21]. A similar report in the literature on a three-dimensional graphene/nickel structure showed an enhancement in thermal conductivity from 0.2639 to 2.6549 W/m K [22]. 

BaSO_4_ is widely used for the production of alloys, cement, ceramics, and glass as a luminophoric material or pigment in various coatings [23,24]. Furthermore, BaSO_4_ powder works as the carrier in the thermal control coating for space vehicles [25]. It has a high reflectiive capacity in the wavelength range of 200–1800 nm [26]. For comparison, the boron nitride (BN)–epoxy composite has enhanced thermal properties 2.85 times greater than those of the neat epoxy with 0.57 W/m K [27].

In this study, we report the results of studies on rGO–B–epoxy composites used to coat 7205 aluminum alloy to improve its corrosion resistance and thermal properties. The use of corrosion-proof coatings is a significant method to increase the lifetimes of metal applications. The corrosion resistances of different rGO–B–epoxy composites were investigated using a voltammeter on samples immersed in 3.5 wt% synthetic seawater (ASTM D 1141-98, where the composition with 58.49 wt% NaCl, 26.46 wt% MgCl_2_·6H_2_O, 9.75 wt% Na_2_SO_4_, 2.765 wt% CaCl_2_, 1.645 wt% KCl, 0.477 wt% NaHCO_3_, 0.238 wt% KBr, 0.071 wt% H_3_BO_3_, 0.095 wt% SrCl_2_·6H_2_O, and 0.007 wt% NaF) to simulate a seawater environment. TAFEL analyses were conducted to estimate the anti-corrosion properties of the rGO–B–epoxy composites. 

The thermal property was studied by using the rGO–B–epoxy composites to coat the surfaces of aluminum alloy specimens. The thermal conductivity coefficients of the pure epoxy resin and graphene–ceramic–epoxy composite were compared using the laser flash method.

## 2. Experimental Methods

### 2.1. Materials

A commercial curing agent (CAS #135108-88-2, Henan Tianfu Chemical Co., Ltd., Zhengzhou, China) was mixed into a bisphenol-A (BPA) epoxy resin (Nanya epoxy resin NPEL-128, Taipei, Taiwan), which then formed a cross-linked structure through the ring-opening polymerization (ROP) of the epoxy groups. This was then used to apply a pure epoxy coating on a 1 mm-thick aluminum alloy (7205 alloy) specimen with a 1 hp air compressor connected to a nozzle with a 100 mL container. The chemical composition of the 7205 alloy is listed in Table 1. 

The graphite used in this study was purchased from Sigma-Aldrich Co. (St. Louis, MO, USA). Hammer’s method was used to synthesize the graphene using potassium permanganate (KMnO_4_, 99%), sulfuric acid (H_2_SO_4_), nitric acid (HNO_3_), hydrogen peroxide (H_2_O_2_), and hydrochloric acid (HCl) purchased from Sigma-Aldrich.

Hammer’s method was modified for the graphene synthesis in this study as follows. First, 2.0 g of graphite was added to a mixture of KMnO_4_ (10 g), concentrated H_2_SO_4_ (72 mL), and concentrated HNO_3_ (65%) for an oxidation reaction and stirred for 40 min in an ice bath. Then, the residual slurry was washed with 12 mL of H_2_O_2_ solution (65%) after washing with 400 mL of deionized (DI) water multiple times (more than three times), until the solution was close to neutral (pH ~7). Finally, graphite oxide (GO) was peeled off through ultrasonic vibration. The GO nanosheets (80 mg in 10 mL of DI water) and ammonia water (70 mL) were mixed in an appropriate ratio, and the solution was placed in a hydrothermal tank and heated to 200 °C for 12 h. Then, the solution was repeatedly washed with 0.1 M HCl, DI water, and alcohol, and centrifuged several times, until the solution was close to neutral. Finally, the resultant product was dried in a vacuum oven at 60 °C for 24 h. Dried reduced graphene oxide (rGO) was produced [22].

### 2.2. Method for rGO–B–Epoxy Composites

After adding 45 wt%, 25 wt%, and 12.5 wt% BaSO_4_ into 100 mL of the epoxy resin separately, the B–epoxy composites were produced by gradually adding the dry rGO with 5 wt% and 2 wt% into the 12.5 wt% and 25.0 wt% B–epoxy mixtures in the fast stirring, respectively. The hardening agent with a volume ratio of approximately twice as much epoxy resin was gradually and simultaneously added to the above-mentioned rGO–B–epoxy resins. Then, different amounts of the rGO–B–epoxy pastes were dispensed using an ultrasonic dispenser for 10–20 min. The rGO–B–epoxy composite coatings were applied using a one-horse power air-compressor paint sprayer. The thickness of the composite coatings was approximately 40 µm with two layers of 3M scotch tape used to control the thickness of the rGO–B–epoxy on the surface of the 7205 alloy. They were cured in an 80 °C oven for 4 h, then at 120 °C for 6 h. 

### 2.3. Characterization

#### 2.3.1. The Surface Characterization with SEM and TEM

Transmitting electron microscopy (TEM, JOEL FEM ARM 200F, JEOL Ltd., Tokyo, Japan) was used to analyze all the specimens. Dual-column ultra-high resolution field emission scanning electron microscopy (SEM, FEI Nova 200, Thermo-Fisher Scientific Inc., Waltham, MA, USA) was used for the preparation of the TEM specimens, which had the dimensions of 4 mm × 2 mm and a thickness of 30–50 nm. The rGO–B–epoxy composites were examined using an X-ray diffractometer with a Cu Kα (the wavelength was 1.504 Å, D8 diffractometer, Bruker, Leipzig, German) with a scan rate of 0.2°/s in 2θ range in 20°–60°. 

#### 2.3.2. Raman, FT-IR, X-ray Photon Spectroscopy (XPS), and Atomic Force Microscopy (AFM) for rGO Analysis

The prepared rGO was characterized using vibrational spectroscopy, Raman spectroscopy (BWTek iRaman with a 532 nm laser light source for excitation, Plainsboro, NJ, USA), and Fourier-transform infrared spectroscopy (FTIR, Perkin-Elmer One with ATR kit, PerkinElmer Inc., Waltham, MA, USA) for the I_D_/I_G_ band ratio and the identification of the functional groups. The rGO surface binding energy for the contained elements was determined using a Shimadzu ESCA 750 spectrometer (Shimadzu, Kyoto, Japan) with an X-ray source of Mg Kα (8 kV and 30 mA). The thickness of the rGO was characterized using atomic force microscopy (VEECO Nanoscope 3100 AFM with tapping mode).

#### 2.3.3. Corrosion Resistance

An Altolab potentostat 30 was employed for linear cyclic voltammetry (LSV) examinations conducted to characterize the graphene–epoxy’s corrosion resistance. The 2 cm × 2 cm rGO–B–epoxy composite on alloy 7205 coupons were set into the three-electrode corrosion cell. The electrochemical analysis was conducted by LSV with 3.5 wt% aqueous synthetic seawater at ambient temperature. The working electrode had a 10 mm^2^ corrosion surface area in the electrolyte and a plate corrosion cell from BSA Inc. (Toyama, Japan). A 1 cm^2^ platinum foil was used as the counter electrode, and the reference electrode was Ag/AgCl. The test parameter was controlled by a computerized Autolab PSTAT 30 potentiostat (Metrohm AG, Herisau, Switzerland) with a scan rate of 10 mV/s and scan ranges of −1.0 to +1.0 V (for the B–epoxy composite) and −0.1 to 0.0 V (for the graphene–epoxy coatings). The corrosion potential (*E*corr) and corrosion current density (*j*corr) were determined by the TAFEL equation based on the LSV results.

#### 2.3.4. Thermal Analysis (Laser Flash Method) and Thermogravimetric Analyzer (TGA)

**Figure 1:**
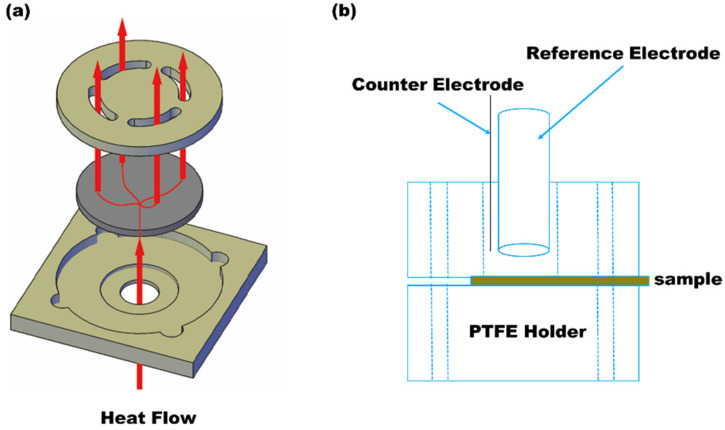
(**a**) In-plane holder for measurement of surface thermal diffusivity and conductivity (from Netsch website) and (**b**) electrochemical test apparatus using in this study (sample as working electrode).

A Netsch LFA 467 HyperFlashTM (NETZSCH-Gerätebau GmbH, Wittelsbacherstraße, Selb, Germany) laser flash thermal analyzer was used. Two sample holders were employed: the 10 mm standard and in-plane holders for the vertical (z-axis) and the xy-plane thermal diffusivities, respectively. The measurements were made at room temperature (25 °C) and ambient atmosphere. The in-plane holder could measure the surface thermal diffusivity, as shown in Figure 1. The laser output voltage was 260 V with 30 ms pulse intervals, and the upper infrared detector was equipped with a focusing lens for data collection. The pulse mode analysis was used for thin and fast-conducting samples. The standard and in-plane thermal diffusivities were obtained for the graphene–epoxy composite coatings. The thermal induction was obtained using laser light at the bottom of the sample (the backside of the 7205 alloy), and the IR detector with ZoomOptics™ of the LFA467 was used to detect the heat flow from the surfaces of the samples.

The thermogravimetric analyzer (TGA Sestsys EVO, Setaram Inc., Caluire-et-Cuire, France) was used to examine the epoxy composite coatings to reveal the additive amounts. A symmetrical hang-down balance was used to continually record the weight changes in the coatings with a ±0.1 μg resolution during the measurements. The temperatures of the samples were controlled and measured with a measurement accuracy of ±0.1 °C. Argon (Ar) gas was purged into the heating chamber at a flow rate of 20 sccm to protect the heating device.

## 3. Results and Discussion

### 3.1. rGO Synthesis and Characterization

**Figure 2:**
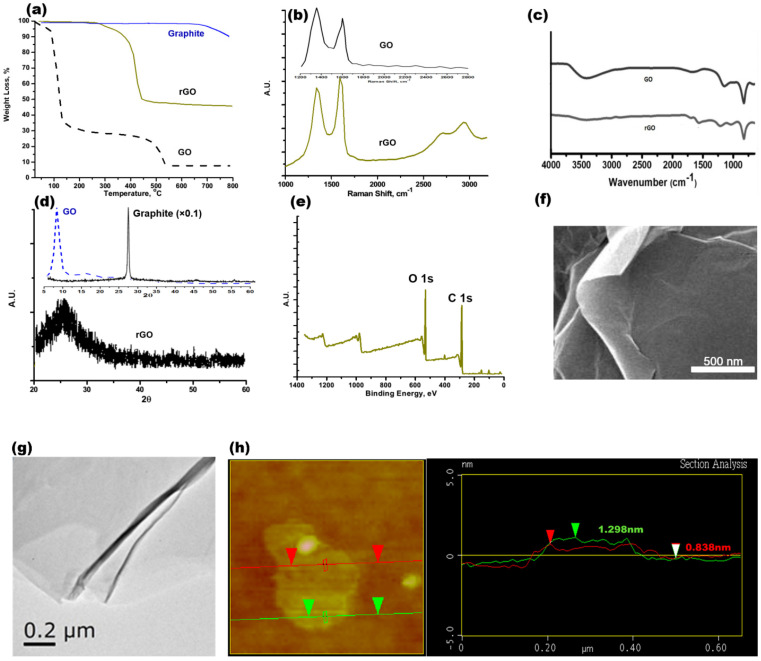
Characterizations of reduced graphene oxide (rGO) produced via (**a**) TGA, (**b**) Raman spectra with 532 nm laser excitation, (**c**) FTIR, (**d**) XRD, (**e**) XPS, (**f**) SEM, (**g**) TEM, and (**h**) AFM.

The characterization results with rGO are shown in Figure 2. The onset and offset temperatures for rGO were approximately 312 °C and 445 °C, respectively, as shown in Figure 2a. Following the oxidation from graphite to GO by Hammer’s method, the oxygen-containing functional groups are estimated to be 64.8 wt%, and the oxygen functional groups’ reduction from GO to rGO by Hammer’s method is about 19.7 wt%. Based on the vibrational spectroscopy analysis, the I_D_/I_G_ band ratio (the D band is at ~1296 cm^−1^ and G band was at~1604 cm^−1^) was approximately 1.04, and the D+G band was approximately 2886 cm^−1^, as shown in the Raman results of Figure 2b. In Figure 2c, the FTIR results show that the rGO had a slight and broadened peak with carboxyl ~OH functional stretching at 3200–3500 cm^−1^. There is C=C sp^2^ stretching for un-oxidized graphite at ~1662 cm^−1^. The alcohol C~OH stretching peak is approximately 1225 cm^−1^. Furthermore, the peak at 1056 cm^−1^ is defined as the C-O-C stretching mode. In comparison to the GO vibrational spectral peaks, the I_D_/I_G_ ratio is approximately 1.21 and the carboxyl ~OH stretching at 3000~3500 cm^−1^ is more obvious in the FTIR spectrum of rGO. 

In Figure 2d, the graphite peak is sharp at 2θ~25°, the GO is a broadened peak at 2θ ~9.6°, and the broadened peak for rGO or graphene is at 2θ–25.2°. The graphite was exfoliated with the high oxidants and reducing agents when using the modified Hammer’s method. The crystalline structure of rGO is not as perfect a crystalline as that of graphite. The XRD pattern should be broader, with a lower intensity, to show the poor crystallinity. In Figure 2e, the XPS results explicitly show the binding energies for elements on the surface of the rGO. The binding energies at 280–290 eV and 524–536 eV are assigned to C 1s and O 1s, respectively. Using the semi-quantification method for the XPS results, the weight percentages for the C and O elements are 64.3 wt% and 33.4 wt%, respectively. Other elements include Cl, S, and Si for the balance of the weight ratio. 

### 3.2. rGO–B–Epoxy Composite Surface Morphology

The SEM examination results for rGO are shown in Figure 2f. Copper tape was used to mount the dried rGO onto the SEM holder. Some brighter areas (for the layer-by-layer boundary and edges of the rGO) could be observed for the rGO flakes, which indicate oxygen-containing functional groups on the rGO surface. In Figure 2g, the TEM image of the rGO shows the layer–layer stacking morphology. The darker area shows a greater thickness of rGO. An AFM analysis with the tapping mode for rGO explicitly showed the different rGO thicknesses of 0.838 nm and 1.298 nm for 2 and 3 layers, respectively, as shown in Figure 2h.

The rGO–B–epoxy coatings were not very smooth like the epoxy-only coatings. The SEM images of the rGO–B–epoxy composites were examined and showed no significant differences. We conducted a TEM analysis after FIB sample preparation in a cross-sectional investigation. The three samples with 0, 2, and 5 wt% rGO added to the B–epoxy matrix were examined.

**Figure 3:**
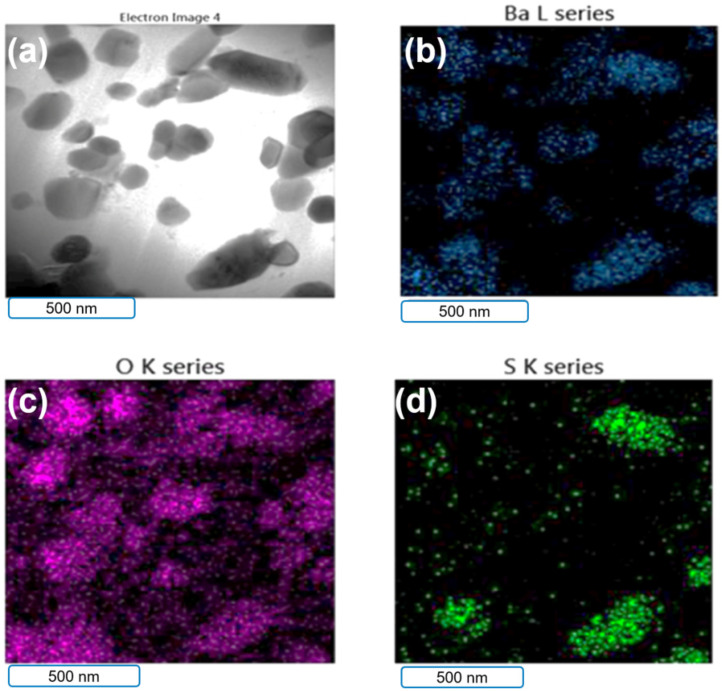
TEM/EDS analysis results for B–epoxy matrix. (**a**) secondary-electron image, (**b**) Ba elements mapping, (**c**) oxygen element mapping, (**d**) sulfur element mapping.

**Figure 4:**
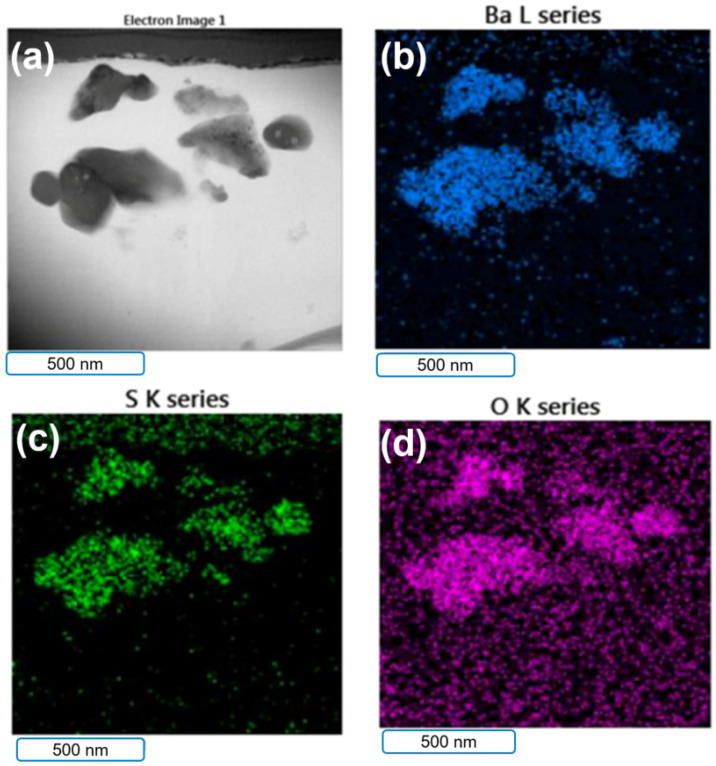
Electron images of 5 wt% rGO–B–epxoy. (**a**) secondary-electron image, (**b**) Ba elements mapping, (**c**) oxygen element mapping, (**d**) sulfur element mapping.

Figure 3 and Figure 4 show the TEM/EDS mapping analysis results for the B–epoxy matrix. The elemental mapping showed that the particles in the epoxy matrix were BaSO_4_. The volume-to-mass ratio of rGO was much larger than those for the epoxy and BaSO_4_. To make better coatings, the amounts of rGO and BaSO_4_ added were approximately 2 wt% rGO with 25 wt% BaSO_4_ and 5 wt% rGO with 12.5 wt% BaSO_4_. The B–epoxy in Figure 3 did not have the rGO morphology, whereas some rGO flakes could be found for the 5 wt% rGO–B–epoxy in Figure 4.

Figure 4 shows an electron image of the coating layer with 5 wt% graphene and 12.5 wt% BaSO_4_. The TEM images for graphene with larger magnification show the typical morphologies. 

**Figure 5:**
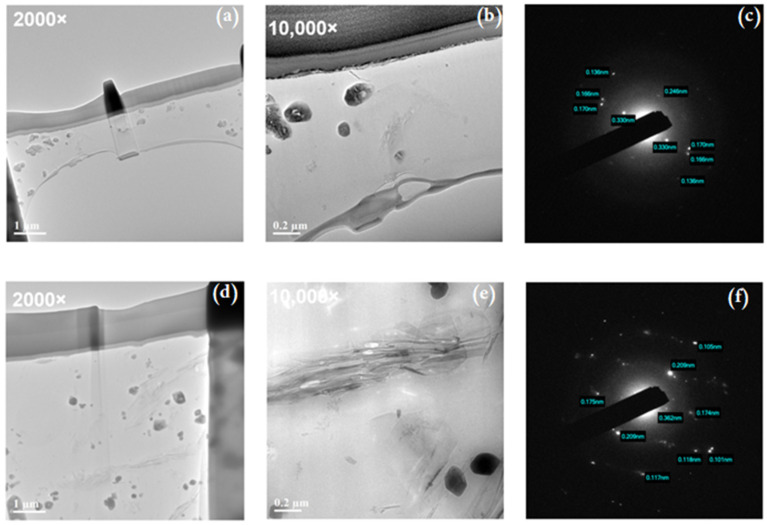
TEM and select area electron diffraction (SEAD) images of 2 wt% and 5 wt% rGO–B–epoxy composites. (**a**) 2 wt% rGO–B–epoxy in 2000 magnification, (**b**) 2 wt% rGO–B–epoxy in 10,000 magnification, (**c**) SEAD pattern for 2 wt% rGO–B–epoxy, (**d**) 5 wt% rGO–B–epoxy in 2000 magnification, (**e**) 5 wt% rGO–B–epoxy in 10,000 magnification, (**f**) SEAD pattern for 5 wt% rGO–B–epoxy.

The TEM images with 2 wt% and 5 wt% rGO-B-epoxy composites. For 10,000 magnification, the rGO layer-by-layer structures could be found in Figure 5b,e. The 5 wt% rGO-B-epoxy is showing the rGO and BaSO_4_ connecting or touching each other, shown in Figure 5d. In Figure 5c,f, the SEAD images for lattice distances of rGO and BaSO_4_. The 0.246 nm and 0.303 nm may be he lattice distances for rGO. Others may be for BaSO_4_.

### 3.3. XRD for rGO–B–Epoxy Composites

Furthermore, select area electron diffraction (SEAD) images of the epoxy–BaSO_4_ and graphene–epoxy composites are shown in Figure 4. The B–epoxy exhibited diffraction dots with d-spacing indications.

**Figure 6:**
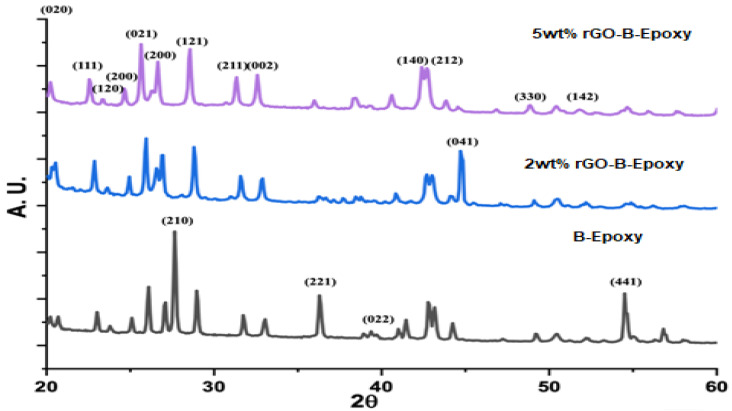
XRD results for B–epoxy and rGO–B–epoxy composites.

Based on the XRD results, the BaSO_4_ was multi-crystalline, and we could find marked facets corresponding to the degree, shown in Figure 6. The assignments of the crystalline phases are marked as the facet numbers in Figure 6 [21]. A comparison of the SEAD and XRD data shows that the BaSO_4_ crystalline facets (041) and (200) were found with both analysis techniques. However, the XRD results do not easily identify the peak for reduced graphene oxide in Figure 6, which normally could be found around 25° as an amorphous sharp peak. The TEM/SEAD analysis results show diffraction dots with d-spacing values of 0.345–0.350 nm for (110), 0.170–0.174 nm for (220), and 0.190–0.192 nm for (211) [28]. Furthermore, carbon crystalline facets at (004) and (002) were also found, which might have been a contribution from the epoxy thermosetting plastic, as shown in Figure 6.

### 3.4. rGO–B–Epoxy Composites for Corrosion Proofing 

**Figure 7:**
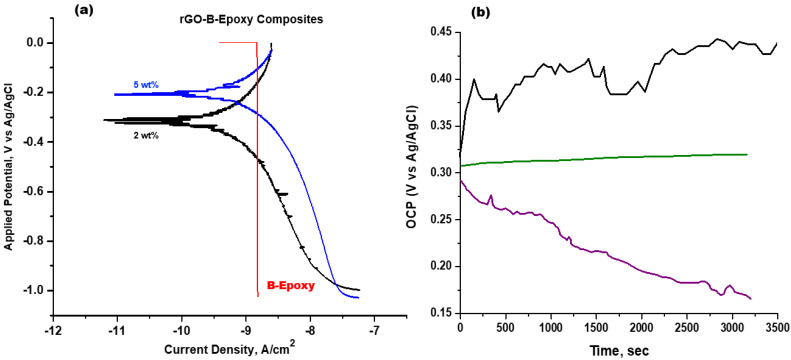
(**a**) TAFEL plot and (**b**) OCP for rGO–B–epoxy and B–epoxy. (black for B–epoxy; green for 2 wt% rGO–B–epoxy, and purple for 5 wt% rGO–B–epoxy).

An experiment to test the corrosion-proofing abilities was conducted in 3.5 wt% synthetic seawater (ASTM D 1141-98) using the electrochemical cell with LSV. The 7205 alloy LSV result shows a negative corrosion potential of approximately −1.0 V (versus the Ag/AgCl reference electrode). For the B–epoxy composite coating, the redox curve could not be found using the LSV result with a TAFEL analysis, as shown in Figure 7. The linear trend for the B–epoxy exhibited an amazing anti-corrosion property under the test conditions. However, we did not have sufficient long-term and true-environment test results to reach a conclusion. The *E*corr and *j*corr values for the 2 wt% rGO–B–epoxy coating on the 7205 alloy surface were −0.3118 V and −9.4694 μA/cm^2^, respectively. The 5 wt% rGO–B–epoxy coating had values of −0.1906 V and −9.3724 μA/cm^2^ for *E*corr and *j*corr, respectively. For an anti-corrosion protective coating layer, a more positive *E*corr indicates better protection. The *j*corr values of the rGO–B–epoxy composites with 2 wt% and 5 wt% rGO added were almost the same. Thus, the 5 wt% rGO–B–epoxy coating was more corrosion-proof than the 2 wt% rGO–B–epoxy coating because of the higher *E*corr. Nevertheless, the B–epoxy coating did not show a redox curve in the TAFEL analysis, which may imply that this coating is the best corrosion-inert layer for certain applications. The 45 wt% BaSO_4_ could extend the epoxy composite lifetime for corrosion applications.

### 3.5. Thermal Properties of rGO–B–Epoxy Composites 

The thermal diffusivities and conductivities at 25 °C for the B–epoxy and rGO–B–epoxy composites are listed in Table 2. The dried powder B–epoxy composite showed standard and thermal diffusivity and conductivity values of 0.179 mm^2^/s and 0.121 W/m K, respectively. When we touched the rGO–B–epoxy coatings in this study with our hands, the surfaces of the coatings felt cold. The thermal diffusivities of the rGO–B–epoxy composites should have been much higher than that of the B–epoxy coating. The in-plane thermal properties (the thermal diffusivity and conductivity) found using the laser flash method for the 2 wt% rGO–B–epoxy coating were 51.46 mm^2^/s and 133.0 W/m K, respectively. The best thermal properties were found for the 5 wt% rGO–B–epoxy composite coating, which had a thermal diffusivity and thermal conductivity of 71.38 mm^2^/s and 165.0 W/m K, respectively. In contrast to the thermal properties found using the standard method (heat flow from bottom to top), the 2 wt% rGO–B–epoxy composite coating had thermal diffusivity and conductivity values of 9.78 mm^2^/s and 25.28 W/m K, respectively. Furthermore, the thermal diffusivity and conductivity of the 5 wt% rGO–B–epoxy were 15.83 mm^2^/s and 36.60 W/m K, respectively. It was impressive that adding 2 wt% and 5 wt% graphene to the B–epoxy could dramatically improve its thermal properties (diffusivity and conductivity).

**Figure 8:**
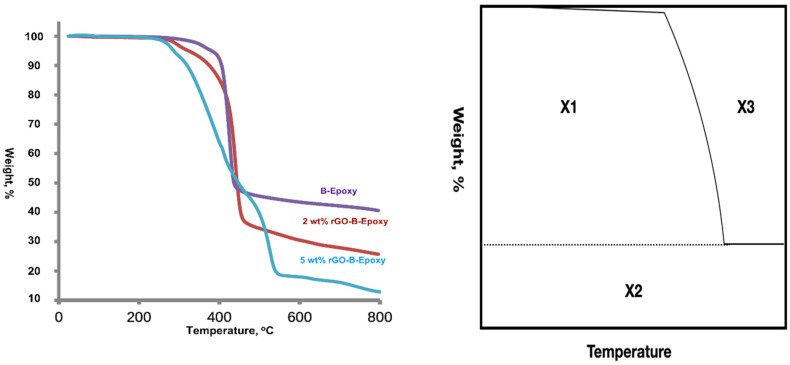
TGA results for epoxy-based composite coatings (**left**) and IDPT method by Doyle (**right**).

The amounts of BaSO_4_ and rGO added to the epoxy resin were calculated. The real amounts added to the coatings were investigated using a thermogravimetric analyzer (TGA). The TGA examination showed that there was approximately 39.7 wt% residual BaSO_4_ in the B–epoxy coating. It was different from the 5.3 wt% used for the preparation of the B–epoxy composite resin. The TGA examinations of the 2 wt% and 5 wt% rGO–B–epoxy coatings showed 11.6 wt% and 26.3 wt% residual BaSO_4_ values, respectively. These represented differences of 0.9 wt% and 1.3 wt% from the values using in the preparation of the B–epoxy resins, as shown in Figure 8. The TGA results could be used with the first-order derivative to show the decomposition temperature. As shown in Figure S1, the 5 wt% rGO–B–epoxy composite coating had three peaks at 284, 409, and 526 °C, which was different from the 2 wt% rGO–B–epoxy composite with one peak at 438 °C, and the B–epoxy composite with one peak at 427 °C. The decreasing zone did not show a dramatic drop for the BaSO_4_–epoxy composite from the TGA results, as shown in Figure 8.

The TGA results could be used with the first-order derivative to show the decomposition temperature. A thermal stability assessment used the integral procedure decomposition temperature (IPDT) by Dolye [29]:

IPDT = A* K* (T_f_ − T_i_) + T_i_ (1)

A* = (X_1_ + X_2_)/(X_1_ + X_2_ + X_3_) K* = (X_1_ + X_2_)/X_1_ (2)

where A* is the ratio between the area subtended by the TGA curve and the total graph area, T_i_ is the initial temperature (25 °C), and T_f_ is the final temperature (800 °C), as listed in Table 2. Calculations show that the IDPT data for the B–epoxy, 2 wt% rGO–B–epoxy, and 5 wt% rGO–B–epoxy composite coatings were 1256.90 °C, 900.86 °C, and 547.25 °C, respectively. The BaSO_4_ contents had the following order: B–epoxy coating (~45 wt% BaSO_4_) > 2 wt% rGO–B–epoxy coating (~26.3 wt% BaSO_4_) > 5 wt% rGO–B–epoxy coating (~11.6 wt% BaSO_4_). A larger amount of BaSO_4_ provided better thermal stability with the ceramic additive as a thermal barrier. The IDPT results were the opposite of the thermal diffusivity and conductivity results, where the 5 wt% rGO–B–epoxy had the best thermal diffusivity and conductivity with the least amount of BaSO_4_ added. In Figure 6, TEM images with a magnification of 10,000 reveal that rGO flakes covered or attached to some of the BaSO_4_ particles. Because graphene has great surface electron conductivity, the thermal conductivity was also great. With the good dispersive preparation of the composite epoxy resin, the rGO–B–epoxy was well connected and had good thermal flow tunnels or channels. The 5 wt% rGO–B–epoxy showed the best thermal properties out of the three samples. This was because of the good dispersion of the BaSO_4_ and graphene in the epoxy resin. The amount of BaSO_4_ decreased with an increase in the graphene added. This was crucial for the ultrasonic dispersive method because the energy transferred through the resin had to be equal and homogeneous throughout the 40 μm coating thickness.

## 4. Conclusions

We reported our recent epoxy-based composites with BaSO_4_ powder and added rGO to enhance the corrosion-proof and thermal properties. The epoxy–BaSO_4_ composite coating was the best corrosion protection coating, beyond the 2 wt% and 5 wt% rGO–B–epoxy composite coatings on the 7205 alloy. Based on the LSV and TAFEL analyses, the epoxy–BaSO_4_ composite was very stable in the corrosion test solution, and no redox curve could be found. The rGO–B–epoxy composites showed good corrosion protection ability, with higher *E*corr values (−0.19 V and −0.31 V for the 2 wt% and 5 wt% rGO contents, respectively) than the 7205 alloy (~−1.0 V). The thermal properties in the vertical direction of the coatings were not very different for the different samples. However, the in-plane thermal properties were very impressive, with values of 160.0 W/m K for the thermal conductivity and 71.38 mm^2^/s for the thermal diffusivity of the 5 wt% rGO–B–epoxy composite coating. The 2 wt% rGO also showed values of 51.46 mm^2^/s and 133.0 W/m K for the in-plane thermal properties. A further study of the thermal conductivity and diffusivity should be conducted on the ultimate compositions of epoxy resin-based composites. 

## Figures and Tables

**Table 1:** Chemical composition of 7205 alloy.

Element	Content (wt%)
Mg	0.499
Si	0.474
Mn	0.025
Cu	0.007
Zn	0.004
Ti	0.028
Fe	0.243
Be	0.004
Pb	0.001
Al	98.50

**Table 2:** Thermal properties of epoxy-based composites on 7205 alloy.

BaSO_4_–Epoxy (A* = 0.8200; K* = 1.9385)	2 wt% Graphene–Epoxy (A* = 0.71389; K* = 1.5831)	5 wt% Graphene–Epoxy (A* = 0.5250; K* = 1.2836)
	Thermal Diffusivity, mm^2^/s	
Standard, 0.179 (±0.002)	Standard, 9.781 (±0.063) In-plane, 51.46 (±3.902)	Standard, 15.83 (±0.071) In-plane, 71.38 (±2.960)
	Thermal Conductivity	
Standard, 0.121 (±0.001)	Standard, 25.28 (±0.160) In-plane, 133.0 (±10.10)	Standard, 36.60 (±0.170) In-plane, 165.0 (±6.800)

## Data Availability

Not applicable.

## Supplementary Materials

The following supporting information can be downloaded at: https://www.mdpi.com/article/10.3390/polym14153144/s1, Figure S1 [30]: The differential thermal gravitometer (DTG) for B-Epoxy (left), 2wt% rGO-B-Epoxy (middle) and 5 wt% rGO-B-Epoxy (right).
