# Supplementary material for: Corrosion Resistance and Thermal Conductivity Enhancement of Reduced Graphene Oxide–BaSO4–Epoxy Composites
Source: Polymers (Basel). 2022 Aug 2;14(15):3144. doi: 10.3390/polym14153144 (PMC9370817; doi:10.3390/polym14153144)
Supplement: Supplementary file 1 [file polymers-14-03144-s001.zip › polymers-1723272-supplementary.pdf]

Supplemental Information

Corrosion Resistance and Thermal Conductivity Enhancement of Reduced Graphene Oxide-$BaSO_4$-Epoxy Composites

Tung-Yuan Yung[1], Wen-Fang Lu[1], Kun-Chao Tsai[1], Jeng-Shiung Chen[2], Kwan-Nang Pang[3], Yu-Chih Tzeng[4], Hsin-Ming Cheng[5*], Po-Tuan Chen[6,*]

1 Nuclear Fuels and Materials Division, Institute of Nuclear Energy Research, Taoyuan 325, Taiwan, R.O.C.; romeoyung@yahoo.com (T.-Y.Y.); wflu@iner.gov.tw (W.-F.L.); tsaijohn@iner.gov.tw (K.-C.T.)
2 Yottadeft Optoelectronics Technology Co., Ltd., Taipei 10460, Taiwan R.O.C.; jsc@yottadeft.com
3 Institute of Earth Science, Academia Sinica, Taipei, 10591, Taiwan R.O.C.; knpang@earth.sinica.edu.tw
4 Department of Vehicle Power System Engineering, Chung Cheng Institute of Technology, National Defense University, Taoyuan 335, Taiwan R.O.C.; a0932467761@gmail.com
5 Department of Electronic Engineering and Organic Electronics Research Center, Ming Chi University of Technology, New Taipei City 243, Taiwan R.O.C.; SMCheng@mail.mcut.edu.tw
6 Department of Vehicle Engineering, National Taipei University of Technology, Taipei 106, Taiwan R.O.C.; r92222019@ntu.edu.tw
* Correspondence: SMCheng@mail.mcut.edu.tw (H.-M.C.); r92222019@ntu.edu.tw (P.-T.C.)

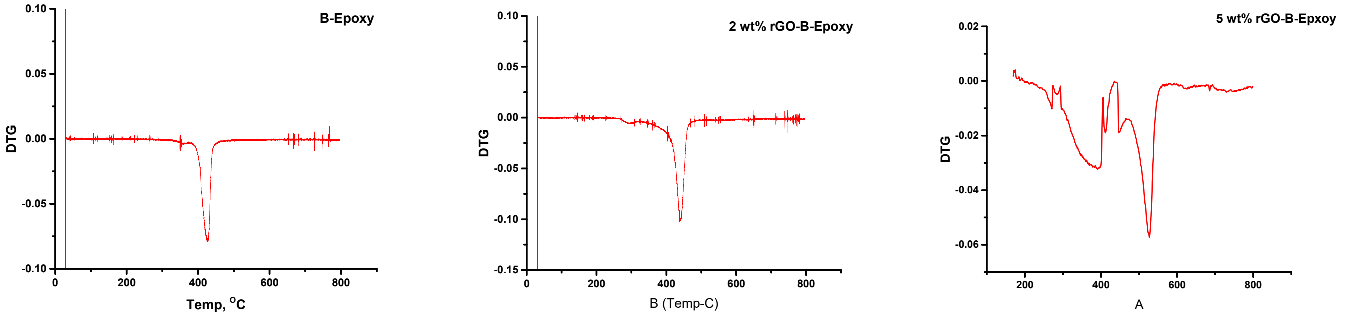

Figure S1. The differential thermal gravitometer (DTG) for B-Epoxy (left), 2wt% rGO-B-Epoxy (middle) and 5 wt% rGO-B-Epoxy (right)

Reference:

[30] Zotti, A.; Zuppolini, S.; Borriello, A.; Zarrelli, M. Thermal and mechanical characterization of an aeronautical graded epoxy resin loaded with hybrid nanoparticles. *Nanomater.* **2020**, 10, 1388.
